# An Experimental Study of Team Size and Performance on a Complex Task

**DOI:** 10.1371/journal.pone.0153048

**Published:** 2016-04-15

**Authors:** Andrew Mao, Winter Mason, Siddharth Suri, Duncan J. Watts

**Affiliations:** 1 Microsoft Research, 641 Avenue of the Americas, New York, NY 10011, United States of America; 2 Facebook Inc., 1299 Pennsylvania Avenue, NW, Washington, DC 20004, United States of America; Northwestern University, UNITED STATES

## Abstract

The relationship between team size and productivity is a question of broad relevance across economics, psychology, and management science. For complex tasks, however, where both the potential benefits and costs of coordinated work increase with the number of workers, neither theoretical arguments nor empirical evidence consistently favor larger vs. smaller teams. Experimental findings, meanwhile, have relied on small groups and highly stylized tasks, hence are hard to generalize to realistic settings. Here we narrow the gap between real-world task complexity and experimental control, reporting results from an online experiment in which 47 teams of size ranging from *n* = 1 to 32 collaborated on a realistic *crisis mapping* task. We find that individuals in teams exerted lower overall effort than independent workers, in part by allocating their effort to less demanding (and less productive) sub-tasks; however, we also find that individuals in teams collaborated more with increasing team size. Directly comparing these competing effects, we find that the largest teams outperformed an equivalent number of independent workers, suggesting that gains to collaboration dominated losses to effort. Importantly, these teams also performed comparably to a field deployment of crisis mappers, suggesting that experiments of the type described here can help solve practical problems as well as advancing the science of collective intelligence.

## Introduction

Teams are fundamental to a wide range of production and problem solving tasks [1–3], many of which are “complex” in the sense that they comprise interdependent sub-tasks [4–6]. In spite of their importance, however, and the considerable attention that has been paid to teams across a range of disciplines, including economics, psychology, sociology, and management science, the factors affecting team performance in complex, realistic task environments remain poorly understood, both in theory and in practice.

With respect to theory, models of collective performance focus on one or two causal factors at a time assuming, in effect, that only these factors vary while all else remains equal. In contrast, real-world tasks are sufficiently complex and multifaceted that many different theories—each making different and potentially inconsistent or even contradictory predictions—may be equally applicable to the same situation, with ambiguous results. For example, a fundamental tenet of economic theory is that division of labor, by allowing workers to specialize, can dramatically increase collective productivity [7]. Workers in teams can also learn from others [8], thereby complementing and accelerating gains to specialization by reducing the need to solve problems independently. All else equal, therefore, the combination of increasing specialization and observational learning would seem to imply that team performance should increase with size for complex tasks. Quite to the contrary, however, equally established theories from psychology [9, 10], economics [11], and management [12, 13] suggest that increasing team size can hurt productivity for a variety of reasons: because workers find it increasingly tempting to free ride on the efforts of others [9, 11]; because the overhead associated with communication increases with the number of individuals whose efforts must be coordinated [9, 12, 13]; or because communication between team members leads to herding [14] and groupthink [10, 15]. In complex task environments, therefore, in which all these conflicting factors may exist simultaneously, the relationship between team size and performance is not well described by existing theory.

Empirical studies, meanwhile, also face challenges in addressing the trade-offs between costs and benefits of group size. One the one hand, observational studies [1, 16, 17] have difficulty identifying the casual effect of group size in the presence of potential confounds such as task type, environment, or management style. On the other hand, lab experiments are generally designed to test specific theoretical hypotheses, and hence usually feature “tasks” that are highly simplified in order to exclude potentially confounding complexities. Classical studies of social loafing, for example, have used either simple physical activities like rope pulling, shouting, and clapping [18, 19], or equally simple mental tasks like solving word puzzles, brainstorming or ranking lists [20–22]. Although they differ in certain respects, these studies are alike in avoiding complex tasks with specialized subtasks, complex forms of organization, performance based incentives, or realistic communication and leadership; yet it is precisely these complexities that are of interest to real-world organizational design and management. Collective problem solving experiments in general also tend to favor simplicity at the expense of external validity, both the classical literature dating to the 1960s and earlier [23–30] as well as the more recent literature on networked experiments [6, 31–34]. Again, the objective of these studies has been to isolate key effects by controlling for confounding complexities, and so although more complicated in terms of design than the classical literature, these experiments still utilize highly stylized tasks, simplistic network structures, and limited modes of communication between team members. As with the theoretical literature, therefore, the existing experimental literature also largely avoids the question of how distinct and possibly countervailing mechanisms jointly impact performance in more realistic settings.

In this paper we introduce a conceptual and practical approach to studying the dynamics of team performance—including its relationship with team size—that encompasses tasks of real-world complexity while also satisfying rigorous standards of experimental control. We emphasize that it is not our objective in this paper to “solve” the problem of collective performance in teams, or even to fully understand the relationship between team size and performance. As we argue later, in fact, our results raise numerous new questions that will require additional experiments, and potentially also new theory, to answer. In other words, the objective of understanding the dynamics of team performance in complex tasks is better suited to a research program than to a single experiment or paper. Nevertheless, it is our objective here to advocate for such a research program and to sketch its potential outlines. To that end, we make three contributions which we describe in more detail in the next section. First we describe a real-world activity, namely crisis mapping, that we argue exhibits a number of useful properties as a model task for studies of collective problem solving. Next we describe an open-source, web-based experimental platform that allows teams of arbitrary size to collaborate on this task in a highly instrumented, controllable environment. Finally we illustrate the usefulness of this platform by describing an experiment in which teams of sizes ranging from *n* = 1 to 32 worked on the crisis mapping task, allowing us to understand how diverse theories of collective action and team performance combine in the context of a complex task.

## Crisis Mapping and Collective Problem Solving

Crisis mapping is an important example of “Digital Humanitarianism” [35] in which groups of volunteers collaborate online to monitor, classify, and map real-time information shared by affected populations in the midst of a humanitarian crisis, typically arising from a natural disaster such as an earthquake or a hurricane. The input to the crisis mapping problem is a stream of data, often in the form of social media reports (e.g. a stream of tweets). The output is an annotated *crisis map*, showing relevant events found in the input stream that are categorized, geolocated, and verified. Client agencies such as the United Nations Office for the Coordination of Humanitarian Affairs (OCHA) use the crisis map to inform decisions about resource allocation, inter-agency coordination, or some other type of humanitarian assistance.

### The Standby Task Force

Crisis mapping originated in 2010 in the aftermath of the Haiti Earthquake, during which a small group of volunteers organized spontaneously to monitor emerging news and social media data, and to categorize and verify reports of damage and casualties in the vicinity of Port-au-Prince. The success of this effort led to the formation of the *Standby Task Force* (SBTF), a digital volunteer organization for collaborative, crowdsourced crisis mapping. Since 2010, the SBTF has deployed on more than three dozen occasions, often alongside other digital humanitarian organizations, in response to natural and political crises in more than two dozen countries and spanning six continents. In past deployments, the SBTF has focused on three main tasks, often undertaken by specialized teams: *media monitoring* (searching Twitter, news feeds, and other social media for relevant crisis events and filtering irrelevant reports), *geolocation* (finding physical locations of reported crisis events), and *verification* (checking the quality and correctness of mapped events). SBTF members typically use a combination of technologies to coordinate their efforts (e.g. Google Docs for collating data, Skype for team chat, and a Ning website for persistent information about the organization), aided by a small number of experienced leaders, who provide training to volunteers, coordinate the overall deployment, and communicate with other relief organizations such as OCHA and local NGOs.

### Typhoon Pablo Deployment

**Fig 1:**
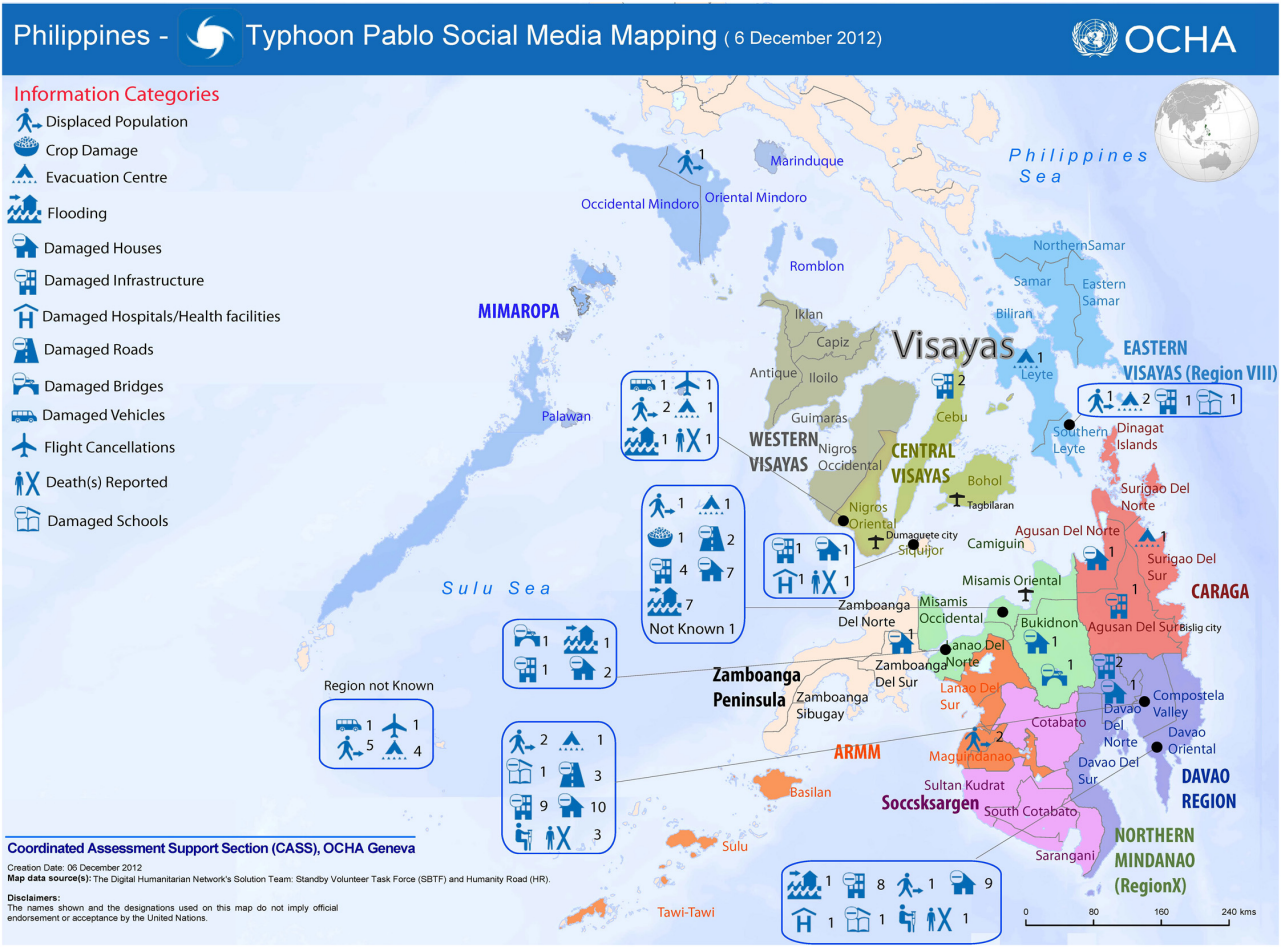
Crisis map produced by the Standby Task Force and Humanity Road in response to Typhoon Pablo, Dec. 6, 2012. Reprinted from http://irevolutions.org/2012/12/08/digital-response-typhoon-pablo/ under a CC BY license, with permission from the Standby Task Force and Humanity Road, original copyright 2012.

Typhoon Pablo (aka Bopha) was a category 5 tropical cyclone that struck the Philippines on December 4, 2012 and caused widespread damage and loss of life over the ensuing 24 hours. On the evening of December 5, in response to a request from OCHA, 32 SBTF volunteers deployed for a 12 hour period with the objective “To collect all relevant tweets about Typhoon Pablo posted on December 4th and 5th; identify pictures and videos of damage/flooding shared in those tweets; geo-locate, time-stamp and categorize this content.” [36] To achieve this goal, they were given a collection of roughly 1600 tweets that had been pre-processed to reduce the number of re-tweets and to preserve tweets with links. Although 32 volunteers registered to work on the task, only 18 distinct user names appear in the resulting list of tagged tweets [37]. It is possible that the unlisted volunteers contributed in other ways (e.g. by verifying existing reports); however, it appears likely that at least some did not contribute in which case the effective group size was between 18 and 32. Over the course of the 12 hour deployment this group identified 93 tweets as relevant; however, some of these tweets reported similar damage in similar locations, hence there were fewer than 93 distinct events [38]. During this same period *Humanity Road*, a different crisis mapping organization, tagged an additional 40 tweets from a separate set. The combined set of 133 tagged tweets was then mapped, and the resulting crisis map submitted to OCHA (see Fig 1).

### Crisis mapping as a model task

Although crisis mapping is just one of many possible collaborative problem solving tasks, we argue that it has a number of advantages for experimental study. First, crisis mapping qualifies as a *complex* task in that it comprises multiple, partially interdependent subtasks. On the one hand, the work of classifying many events can be partially parallelized by assigning different events to different workers. On the other hand, complete parallelization is inefficient as events are often reported many times, where different reports may contain incomplete or inconsistent information. Coordination between workers is therefore helpful for reconciling different versions of the same event, avoiding redundancies, and identifying errors. Coordination can also help ensure that specific subtasks—say filtering, classification, and geolocation—are allocated to individual workers who are most suited to perform them. As with other complex tasks, therefore, crisis mapping benefits both from the division of labor and also coordination between individual workers. Second, however, crisis mapping is nonetheless simple enough that new participants can contribute without any specialized skills ex ante—hence it is possible to recruit subjects from an inexperienced and undifferentiated subject population. Third, crisis mapping is a task that is natively online, and thus experiments conducted in an online environment with subjects recruited from online crowdsourcing sites bear a close resemblance to the real-world activity. Finally, crisis mapping closely resembles other information processing and collective sensemaking activities such as document classification, real-time reporting, or intelligence gathering—hence we anticipate that lessons learned about how teams organize to perform crisis mapping can be generalized at least to other members of this class. Aside from its intrinsic humanitarian interest, therefore, we propose that crisis mapping is also a plausible candidate task for studying the more general phenomenon of collective problem solving in teams.

## The CrowdMapper Platform

**Fig 2:**
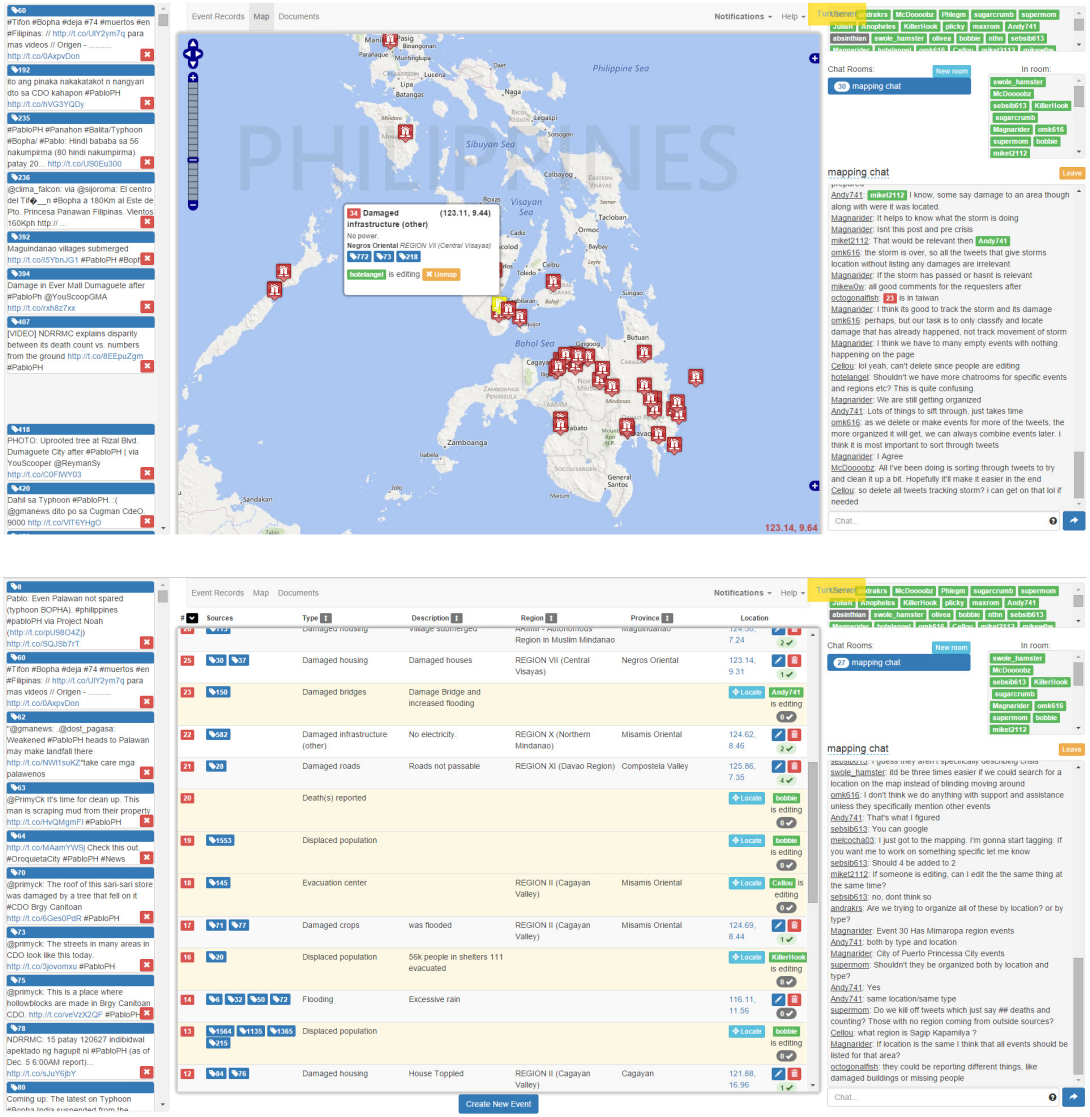
Map (top) and table (bottom) views of the open-source CrowdMapper platform. (Bing Maps imagery reprinted under a CC BY license with permission from Microsoft Corporation).

To replicate the critical features of the crisis mapping task while also maintaining a high level of experimental control, we designed and built a collaborative, real-time web application called *CrowdMapper* that allows many groups of users to work together simultaneously. The application, shown in Fig 2 (see https://youtu.be/xJYq_kh6NlI for a video example) for a single group of *n* = 32, comprises the following components:

- **Event Table**. An editable table in which workers can record crisis reports. Columns include the event type (e.g. washed-out bridges, flooded roads, etc.), a textual description, and location information. Workers can also “vote up” existing reports to signal approval of their accuracy. See the middle part of Fig 2, bottom.
- **Interactive Map**. A map view of the events displays any event geographically with a recorded longitude/latitude. See the middle part of Fig 2, top.
- **Tweet stream**. A common feed of Twitter-style messages (tweets), each potentially containing relevant crisis data including links to outside websites. Workers can click links to view their content, can hide (filter) an irrelevant tweet by clicking on a red **X** button, or can drag a relevant tweet to an existing event report. See the left part of Fig 2.
- **Chat rooms**. Workers can create any number of chat rooms with names of their choosing, and can invite others to join a particular chat room. A tagging syntax allows messages to reference specific workers, tweets, or events, generating clickable links that jump to and focus on the tagged item. See the right part of Fig 2.
- **Documents**. Workers can create any number of documents to record instructions or other persistent data (not shown).

CrowdMapper exhibits a number of advantages as an experimental platform for studying the collective behavior of groups. First, the framework is flexible, allowing for potentially different configurations corresponding to different experimental designs. In the experiment reported here, for example, we were interested specifically in self-organization among teams of different sizes, hence the configuration we used did not prescribe any particular task to participants; each worker was free to use any part of the interface, or even do nothing at all. In other configurations, however, an experimenter might assign particular workers to particular roles, prescribe arbitrary patterns of communication or organizational structure, vary the difficulty of the task (e.g. by increasing or decreasing the number of tweets or available time), or study other types of information gathering tasks. Second, CrowdMapper fully exploits the advantages of online “virtual lab” environments, accommodating many more simultaneous participants than is feasible in existing physical labs, allowing arbitrary treatments to be run in parallel, and facilitating the collection of fine-grained, real-time data about individual and collective behavior. Third, all the code for running experiments like those we describe here is freely available online at http://github.com/mizzao/CrowdMapper. In particular, *CrowdMapper* is built on a newer version of *TurkServer* [39], a web-based behavioral experiment framework, hence other researchers may replicate our results or conduct variations of our particular design.

## The Crisis Mapping Experiment

We examined the relationship between team size and performance by simulating a real-world crisis mapping scenario: specifically the Dec. 2012 Typhoon Pablo deployment of the SBTF. Importantly, our experiment used a set of 1,567 pre-filtered tweets that were also processed by the SBTF in their actual deployment, allowing us to benchmark the performance of our experimental teams against external reality (see S1 Text for discussion of some differences between the experimental and real-world settings).

### Subject recruitment and training

We recruited workers from Amazon’s Mechanical Turk, a crowdsourcing site that is commonly used by researchers to recruit and pay subjects for behavioral experiments [40]. First, we recruited almost 1,300 workers to build a panel for the experiment. During this recruitment task, each worker completed a short, interactive tutorial that explained each part of the interface and required them to map a few example crisis events (see S6 Text for the full text of the tutorial). After completing the tutorial, we collected feedback about any part of the interface that was confusing, as well as workers’ availability by timezone to schedule the simultaneous part of the experiment. We also asked if workers would agree to be contacted for scheduled experiment sessions. Finally, participants were required to affirm (by checking a box) that they were over the age of 18, that they had read and consented to the terms of service (See S5 Text for the full text), and that they understood the potential risks of participating in the experiment. Without actively checking the box they were unable to enter the experiment (see S6 Text for the full text of the statement). The Microsoft Research ethics review committee (OHRP parent organization number IORG #0008066, IRB #IRB00009672) reviewed this informed consent procedure and approved it, along with the overall experimental protocol.

**Fig 3:**
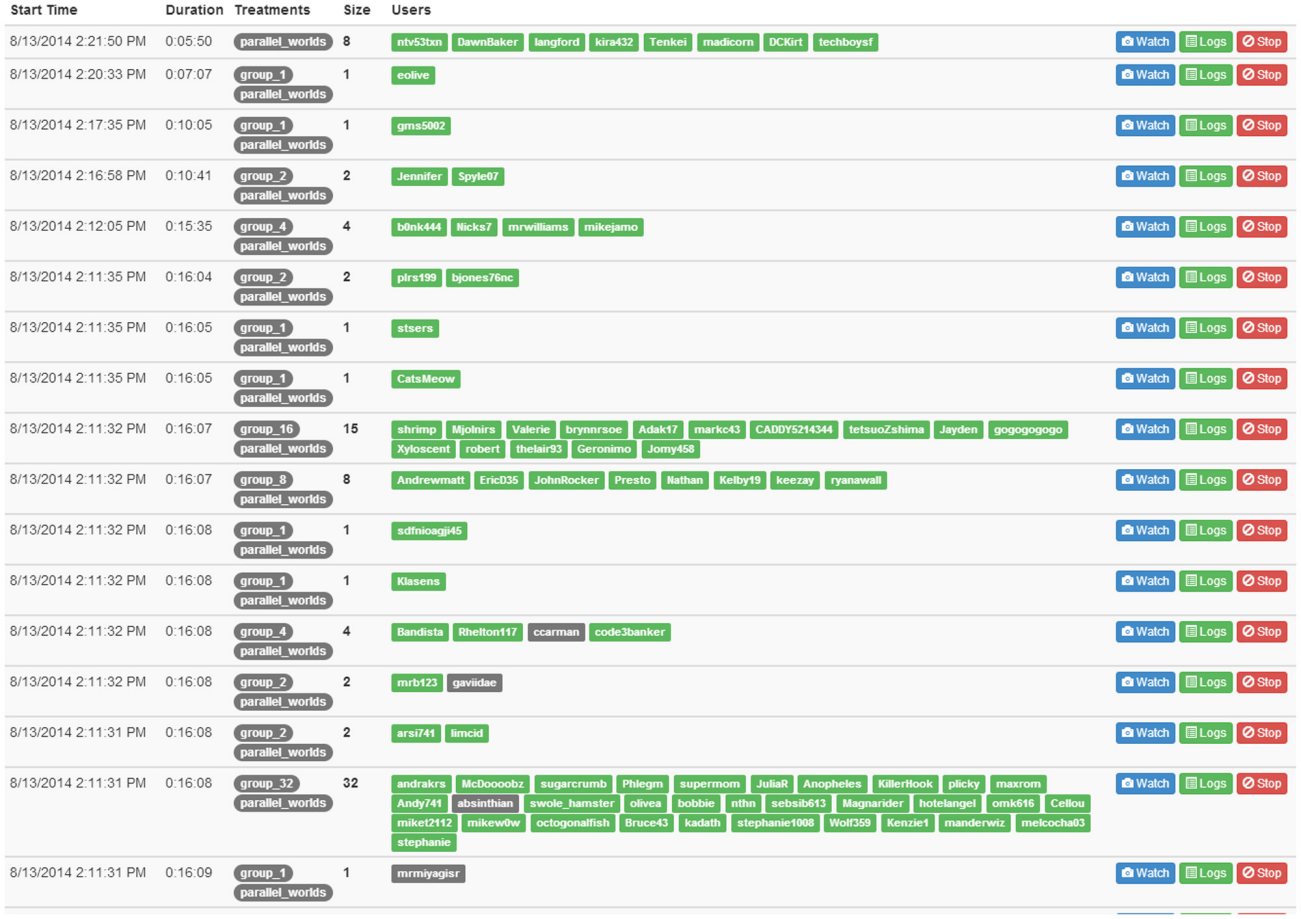
Randomized assignment of approximately 100 workers to different teams in one experiment run, as shown in *TurkServer* [39].

The experiment was conducted over five one-hour sessions: one each on the days Aug. 11–14, 2014 and one on Aug. 25, 2014. For each session, we selected a random subset of workers in our panel who had not yet participated in the task, chose a time of day that showed the most availability by workers’ preferences, and sent an e-mail in advance asking workers to arrive at that particular time. All workers who arrived again completed the same tutorial as they had during recruitment, and again consented to participate in the experiment. Because participants did not arrive at precisely the same time and because they also took varying lengths of time to complete the tutorial, they were assigned to a virtual “lobby” until sufficiently many participants were available to begin the experiment. At this point, all participants in the lobby were released simultaneously and randomly assigned to a predetermined set of teams of sizes *n* = 1, 2, 4, 8, 16, and 32 (see Fig 3 for an example). We studied a total of 47 teams comprising 258 unique individuals, where no individual participated more than once (See Table 1). We did not observe any systematic differences between either participants or team performance on different days. In addition, all participants were asked (but not required) to complete an exit survey in which they could register any complaints about the experiment or the instructions. We did not receive any serious complaints about either.

**Table 1:** Number of teams and participating individuals assigned to each condition. A Fisher exact test on the dropout rate is insignificant at the 10% level across all pairs of conditions, suggesting that the attrition rate did not significantly change based on the treatment.

Team Size	Completed Teams	Number of Individuals	Dropouts (rate)
1	18	21	3 (14.2%)
2	11	22	1 (4.5%)
4	6	24	3 (12.5%)
8	4	31	2 (6.5%)
16	4	59	3 (5.1%)
32	4	123	10 (8.1%)
Total	47	280	22

### The Experiment

Once in the experiment, each team was presented with the same set of 1,567 tweets (i.e. teams only differed in the number of participants assigned to the task). All workers were instructed that they had to complete the following tasks: (1) identify tweets that referred specifically to instances of typhoon-related damage (e.g. washed-out bridges, flooded roads, damaged crops or buildings, displaced population); (2) create event records for every such instance, describing the event in words, attaching the relevant tweets, classifying it as one of several predefined types, and establishing its geographical location (by latitude and longitude as well as province and region); and (3) verify and, if necessary, correct the information in existing event reports. Workers assigned to the *n* = 1 condition were told that they would be working alone; in all other conditions they were told the size of their team, shown the usernames of their coworkers, and instructed on how to create chat rooms for the purpose of communicating; however, they were not given any instructions in how to coordinate. All teams were given one hour from the arrival time of the first members to identify as many crisis-related events as possible, and workers were informed that their individual compensation would vary between a minimum of $6 and a maximum of $15 and would be computed as a joint function of their own time spent working and the overall performance of their team relative to other teams. Specifically, each team was assigned a performance-based hourly wage between $6 and $15, computed as $\text{Team}\;\text{Wage}=\$6+\$9\times \frac{\text{Team}\;\text{Performance}}{\text{Best}\;\text{Team}\;\text{Performance}}$, where we note that the minimum of $6 per hour was considered a norm for the minimum acceptable wage on Mechanical Turk at the time of the experiment. Each participant was then paid an amount corresponding to their team’s wage, scaled by their *own* activity time: Individual Payment = Team Wage×Active Time. Workers were clearly informed of this compensation scheme, and were told that their activity would be monitored (see S2 Text for more details). Individuals could therefore realize the maximum possible payment only by both participating throughout the task and also ensuring that their team performed well, a point that we emphasized during the tutorial. Moreover, because our compensation scheme was based on a combination of individual time worked and collective performance relative to (unobserved) other teams, it was extremely hard to game (i.e. it did not reward any specific type of activity over others), and also penalized both explicit loafing as well as more indirect forms of free riding (e.g. performing “busy work” that didn’t contribute to the stated goals). Workers therefore had real and meaningful monetary incentives both to contribute individually and also to cooperate with their coworkers.

## Results

**Fig 4:**
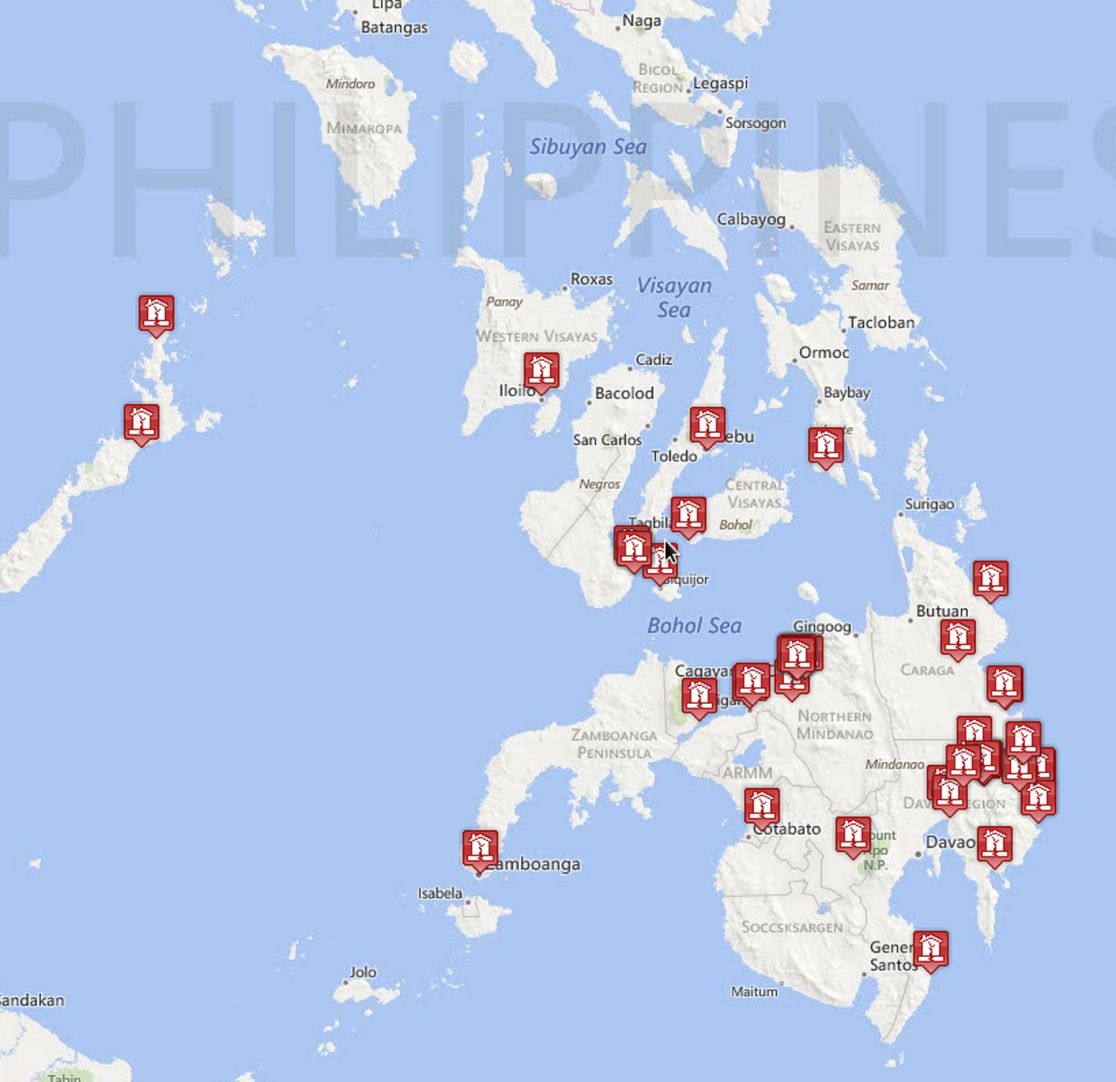
The gold standard crisis map. Bing Maps imagery reprinted under a CC BY license with permission from Microsoft Corporation.

To evaluate team performance, we initially constructed a “gold standard” crisis map as follows. First, we aggregated all of the events generated by all teams in the experiment, and removed all duplicates, ensuring that each potential event was included only once. Second, we then manually checked all the de-duplicated events, and deleted any that could not be independently verified either by directly inspecting linked content or cross-referencing them with the SBTF map. Following this procedure, our gold standard map comprised 49 distinct events reporting specific instances or types of damage at a village or district level, which were referred to by 245 distinct tweets (w focus on events rather than tweets on the grounds that the goal of crisis mapping is to correctly identify as many relevant events as possible for consumption by a client agency such as OCHA). The resulting map is shown in Fig 4 and is available in our software repository at http://github.com/mizzao/CrowdMapper. We note that our gold standard could be missing real events that were not detected by any of our experimental teams, hence we do not refer to it as a “ground truth” map; however, it is by definition impossible for any team to perform better than the gold standard.

### Performance Metrics

For every team *i* we computed three standard measures of performance: *precision*, defined as the fraction of events identified by team *i* that matched the gold standard; *recall*, defined as the fraction of all events in the gold standard that team *i* correctly identified; and *F*_1_ score, defined as the harmonic of mean of precision and recall (see S3 Text for details). Because both precision and recall are important for our application—i.e. both false positives and false negatives impair the utility of a crisis map—we focus on *F*_1_ as our preferred measure of performance, although for explanatory purposes we also include results for precision and recall.

### Performance over time

**Fig 5:**
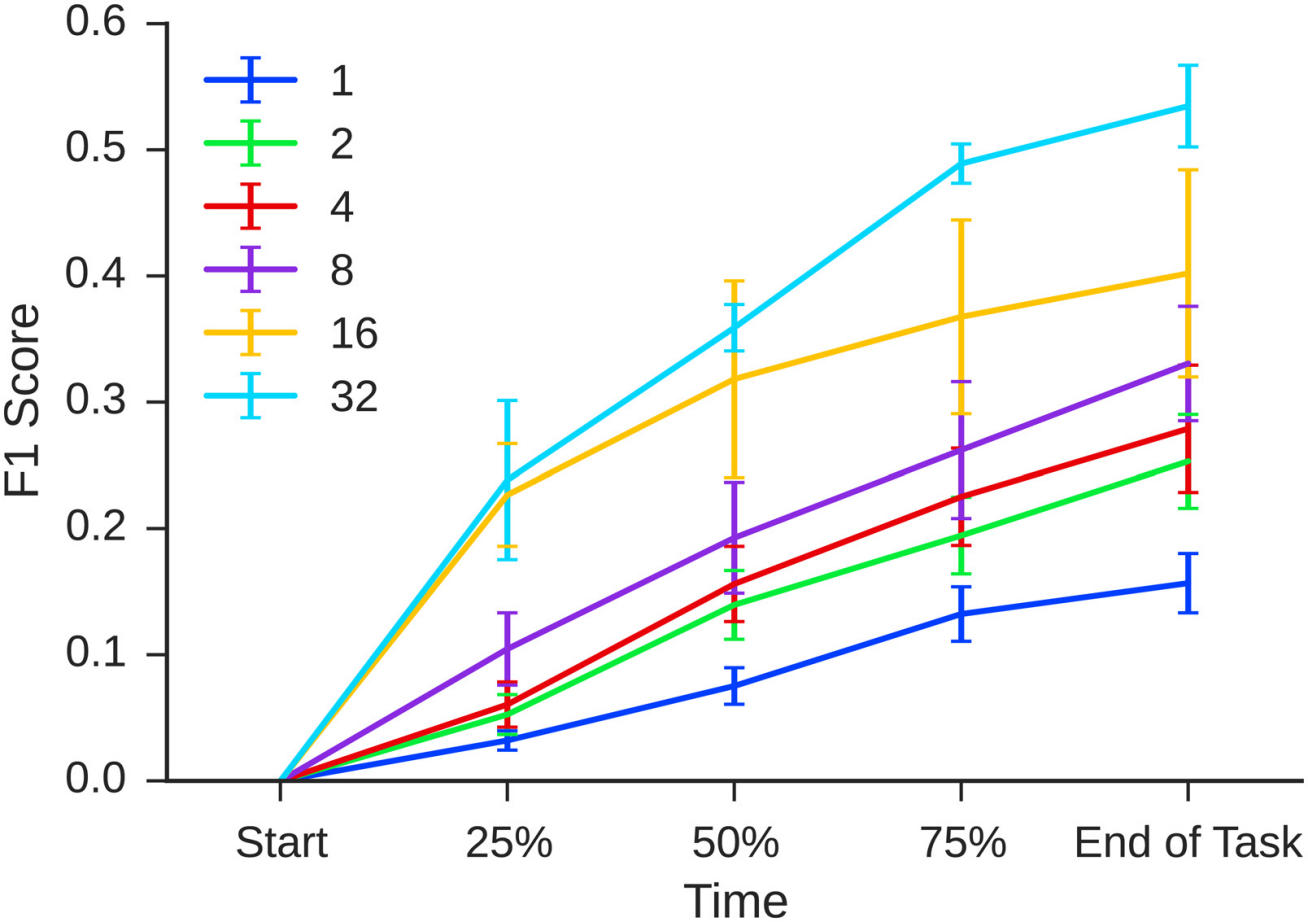
Performance (*F*_1_ score) vs time for teams of nominal size *n* = 1, 2, 4, 8, 16, 32. Error bars show standard errors.

Before proceeding to our main results, we first checked that the teams performed in sensible ways over the course of the experiment. To do this, we reconstructed the exact state of teams at various points in time, allowing us to compare teams at different stages of progress as well as at the end of the experiment. Specifically, we divided the total amount of person-time spent on the task into quartiles and computed statistics after the completion of each quartile. Fig 5 shows performance (*F*_1_ score) over the course of the experiment for teams of all sizes. Performance increased monotonically with time for all team sizes, suggesting that teams were actively engaged in the task, continually adding new event reports (thereby improving recall) and editing existing reports (thereby improving precision). Fig 5 also shows that rank-ordering of performance by team size remained unchanged over the course of the experiment, hence we can focus on the end of the task with little loss of generality.

### Performance of the SBTF

Next, in order to test the external validity of our results we also evaluated the accuracy of the Dec. 2012 Standby Task Force deployment by scoring the spreadsheet of tagged tweets from Typhoon Pablo [37] with the same gold standard map used to evaluate our experimental teams. To do this, we first removed all empty rows and obvious duplicates, as well as tweets tagged by Humanity Road (which used a different dataset), leaving a set of 87 completed or partially tagged tweets. (We note that the spreadsheet used by the SBTF was organized around tweets whereas the reports produced by the teams in our experiment were organized around events. Because many tweets could potentially refer to the same event the 87 tweets classified by the SBTF corresponded to many fewer events, especially in heavily damaged areas.) We then translated this set of tagged tweets using the best possible matches for type, region, and province, and location into the same categorization of data used in CrowdMapper, preserving any partially complete or missing data. This process produced a similar format of data as the teams in our experiment, including event reports that were potentially missing a location or other fields. Finally, we scored this set of event reports using the same procedure as the teams that participated in our experiment. (See, however, S1 Text for some differences between the SBTF deployment and our experiment that could have affected scoring.)

### Computing Person Hours

Although ideally our design required all teams to be fully populated for the entire duration of the experiment, the variance in arrival and tutorial-completion times would have required long waits for some participants, thereby increasing the likelihood of attrition. To address this concern, we released the waiting room no later than 10 minutes after the first arrival, and then continued to assign subsequent arrivals to teams for several minutes after the task started (the slowest arrivals were all routed to a single ‘buffer’ team and this data was excluded from our analysis). As a consequence of this, as well as inactivity of some workers, not all teams had a full complement of workers for the whole hour (see Table 1). A team of nominal size *n* = 32, for example, would almost certainly be missing at least some of its workers for at least some of the time, and because this amount could vary from day to day or even across teams within the same session, two teams of the same nominal size might have substantially different amounts of actual labor available to them. For this reason, we hereafter avoid comparison in terms of nominal team size, instead comparing team performance as a function of “person-hours” $\omega_{p}=\sum_{j=1}^{n}T-t_{j}^{*}-\psi_{j}$, where *T* is the duration of the experiment, $t_{j}^{*}$ is the start time of the *j*^th^ worker, and *ψ*_*j*_ is their “inactive” time. In this way we can compare teams of all sizes in a consistent and meaningful manner.

### Performance vs. Size

**Fig 6:**
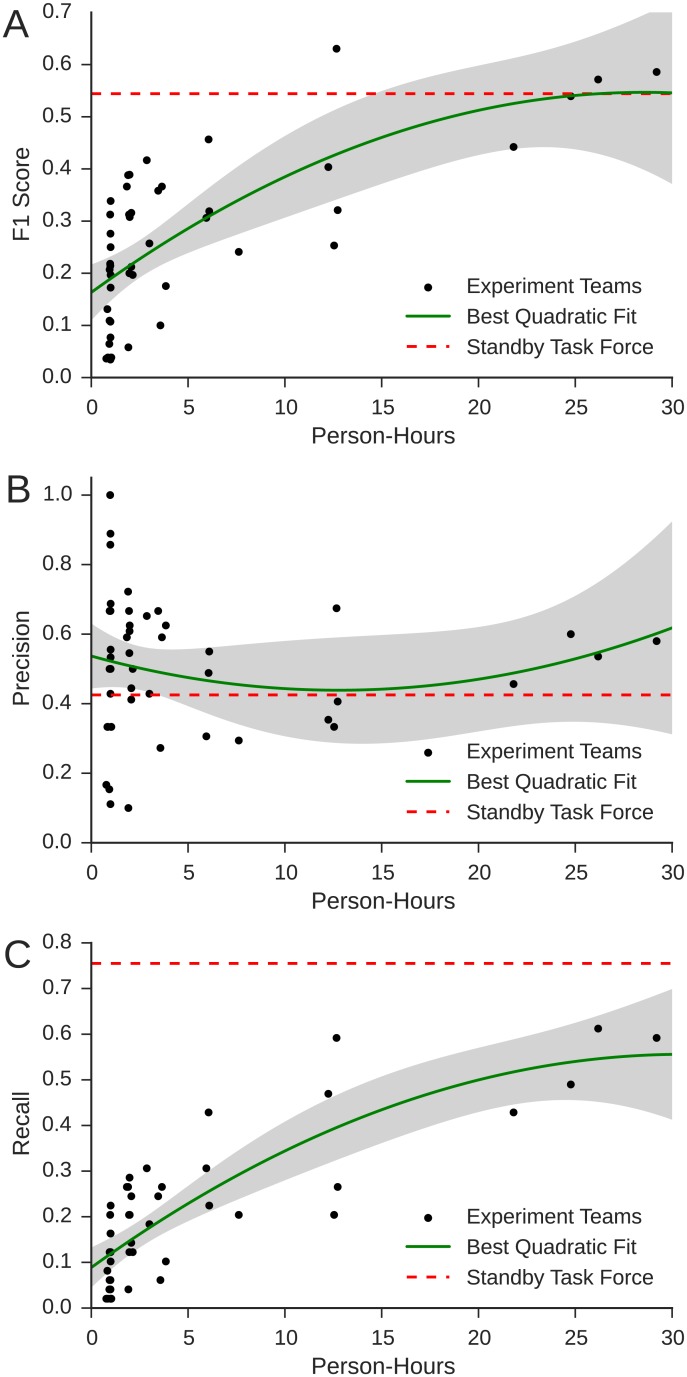
A: *F*_1_ score; B: precision; and C: recall vs. actively worked person-hours. Each dot represents one team, and solid lines indicate least-squares best fit. Dashed lines indicate the performance of the SBTF, and the shaded area shows the 95% CI of the regression mean.

Moving now to our main results, we fit both linear and quadratic models of the form

$$\begin{array}{l} y\sim \beta_{0}+\beta_{1}\omega_{p}+\epsilon \\ y\sim \beta_{0}+\beta_{1}\omega_{p}+\beta_{1}\omega_{p}^{2}+\epsilon \end{array}$$

respectively, where *y* represents team performance and *ω*_*p*_ is person-hours. The quadratic model fit the data well (*R*^2^ = 0.498, *p* < 0.001) and was chosen over the linear model using both BIC and AIC criteria (AIC linear: −66.84, quadratic: −67.02). Fig 6 shows the maximum likelihood fit of the quadratic model (solid line), where the shaded area is the 95% confidence interval of the model, and the dots show the performance of each of the experimental teams (the dashed line represents the performance of the SBTF). Together, the model analysis and Fig 6 reveal three main results (see S4 Text for full details of the quadratic model).

First, as can be seen in Fig 6A, the coefficient for the linear term (*β*_1_ = 0.726, *p* < 10^−7^) is large, positive and highly significant, confirming that average performance increased with person-hours. We emphasize that this result was not ex ante obvious. Prior to participating in the experiment, the workers’ experience with crisis mapping was limited to completing the tutorial. Workers also received no specific instructions on how to perform the task successfully or how to coordinate with one another. Informal reviews of the chat logs and exit surveys confirm that many workers were initially confused about how to proceed in general and frequently asked each other for help in completing specific actions. Under such circumstances, it is entirely plausible that for teams larger than a particular size, additional workers would create more confusion and coordination overhead than useful labor, thereby yielding negative marginal returns rather than simply diminishing. Precisely such an outcome, in fact, is predicted by “Brooks’ law” [12], which states that adding manpower to a late project makes it later. Corroborating Brooks’ prediction, many workers in the larger teams complained in their exit surveys that there were too many people to coordinate and that smaller teams would have been more effective. That smaller teams were not in fact more effective is therefore somewhat surprising.

Second, consistent with the hypothesis that marginal performance is decreasing (although we note the coefficient is not signficant) the model has a negative quadratic coefficient (*β*_2_ = −0.163, *p* ≈ 0.15). This finding is again visually confirmed in Fig 6A (i.e. the curve is concave), and suggests in turn that per-capita productivity was highest for individuals and decreased with team size. Fig 6B and 6C decompose this effect into precision and recall respectively, showing that performance differences were dominated by improvements in recall, which also increased with diminishing marginal returns, whereas precision neither consistently increased nor decreased with team size. Although as we will show next, this result can be explained in terms of so-called social loafing, we again emphasize that the opposite finding would have been equally plausible; that is, *increasing* marginal productivity on account of workers becoming increasingly specialized and thereby benefiting from “learning by doing.” Indeed, precisely such an effect has been reported anecdotally by the SBTF [35], which began as a single undifferentiated team of unspecialized workers and over time developed a stable division of labor among different specialized teams (e.g. media monitoring, geolocation, verification). It is therefore interesting that increasing marginal returns to specialization did not manifest themselves in our experiment, or else did not have sufficient impact on performance to outweigh the losses in per-capita productivity from other causes.

Third, the largest teams achieved levels of performance comparable to the actual 2012 SBTF deployment (indicated by the dashed line). Once again this result is not obvious—indeed, it is quite remarkable—for two reasons. First, although the number of workers in the SBTF was comparable to the largest teams, they participated continually for twelve hours, whereas the experimental teams had only one hour. And second, unlike in the SBTF deployment workers in the experimental teams did not have access to experienced crisis mappers for guidance and leadership, nor could they benefit from established procedures and divisions of labor. Any organization or expertise that the teams acquired had to be developed over course of the experiment, further diminishing the time available to work on the task itself.

### Social Loafing

Why does per-capita productivity decrease with team size? As mentioned above, a plausible candidate explanation is suggested by the extensive literature on “social loafing” [19, 21]; that is, individuals in groups simply exert less effort than when they are alone. To investigate this possibility, we compute individual effort in terms of *effort-hours*, which we compute as follows. First, we define the *action time* *τ*_*a*, *k*_ for the *k*^*th*^ instance of an action of type *a* as the time elapsed between the completion of that action and the completion of the previous action by the same user. We then compute the average action time $\tau_{a}=\frac{1}{m_{a}}\sum_{k}\tau_{a,k}$ over all instances of action type *a* across all workers in all conditions where *m*_*a*_ denotes the number of instances of action *a*. Finally, we compute each individual worker *j*′ *s* total effort in effort-hours as their number of actions weighted by these average times: *E*_*j*_ = ∑_*a*_ *τ*_*a*_ *m*_*a*, *j*_, where *m*_*a*, *j*_ is the number of actions of type *a* taken by individual *j*. By expressing work in units of effort-hours, we can compare individuals and teams alike in a consistent way, noting also that it is possible for an individual to exert more than one effort-hour in one hour of clock time.

**Fig 7:**
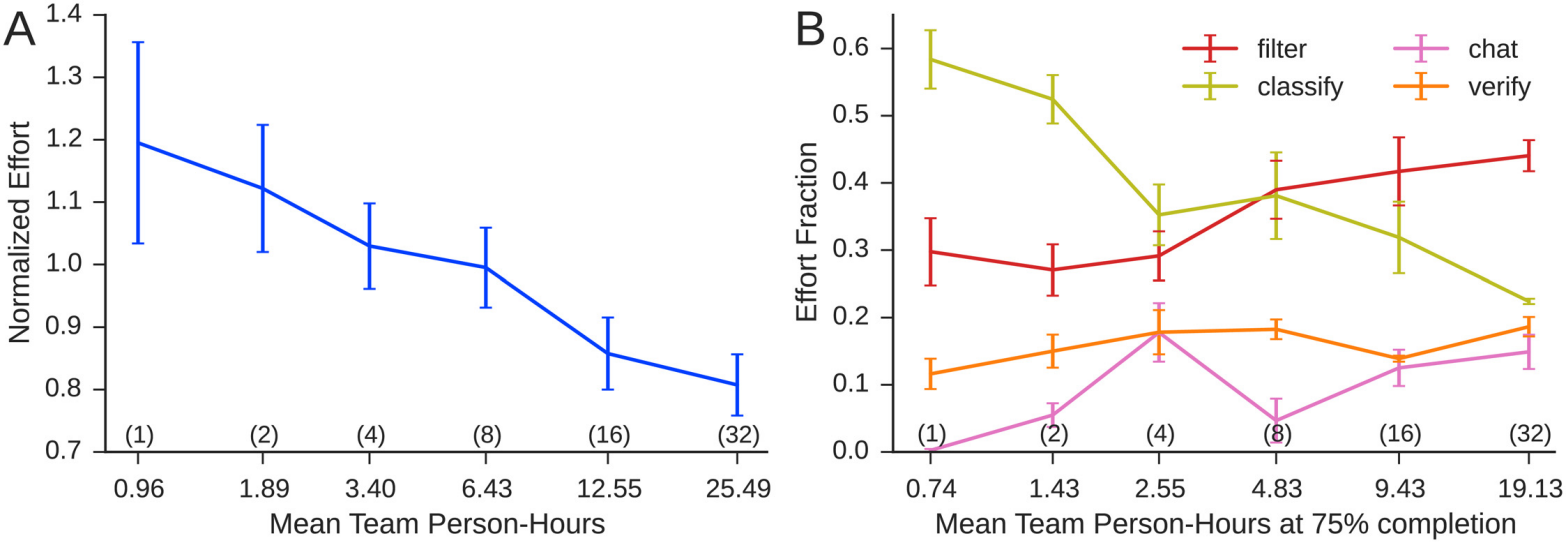
**A**: Individual effort measured in effort hours (see text) as a function of mean person hours for teams of a given nominal size (nominal team sizes are given in parentheses). **B**: Allocation of effort to the four primary task-types as function of final average person hours, evaluated at 75% of final person-hours. We make the comparison at 75% of person hours rather than 100% because one of the groups of *n* = 32 succeeded in filtering all of the nearly 1600 tweets before the experiment completed, hence for the remaining time their computed effort allocation effectively under-counted filtering and displayed more verification. To check for robustness we also computed the same quantities at the end of the experiment. Naturally, the effort allocated to filtering is lower for the *n* = 32 treatment, but otherwise the pattern remains the same. In both panels nominal group sizes are given in parentheses, error bars denote standard error, and *x*-axes are on a log_2_ scale.

Supporting the social loafing hypothesis, Fig 7A shows that average individual effort in the largest groups was roughly 30% less than for independent workers (ANOVA *F*(1, 45) = 1122.3, *p* < 10^−10^). We note, however, that the crisis mapping task allows individuals to choose not only their total amount of effort but also how they allocate effort over different specialized activities. To address this possibility we further divide effort into one of the four high-level action types: *filtering* irrelevant tweets or adding relevant tweets to event reports; *classifying* event reports either in terms of type or location; *verifying* existing information such as by deleting reports, moving tweets between reports, or clicking a report’s “thumbs up (down)” button; and *communicating* with other workers in the chat room(s). Fig 7B shows that individuals in different sized teams did indeed allocate their effort differently: time devoted to classifying events decreased by roughly 50%, while time devoted to filtering tweets increased by roughly the same proportion. We also note that whereas filtering can be as simple as repeatedly clicking the **X** (delete) button on tweets, classification requires more deliberation. The shift from classification to filtering can therefore be viewed as a novel variant of social loafing that arises specifically in complex tasks in which workers can shift their effort between activities of different difficulty.

### Collaboration

In addition to the amount and allocation of effort, we can also investigate the extent that effort is exerted in collaboration with others. Collaboration is potentially important as it has been shown to dramatically improve the performance of teams vis-a-vis equivalent numbers of independent workers [34]. In the context of crisis mapping, collaboration could help performance by reducing both error rates and also redundant effort (individuals reporting the same event multiple times). To quantify collaboration, we define a collaboration index $c\left(e_{il}\right)=2^{H_{il}}$, where *e*_*il*_ refers to the *l*^*th*^ event worked on by the *i*^*th*^ team, $H_{il}=\sum_{j=1}^{n_{i}}-p_{j}\log_{2}p_{j}$ is the standard information-theoretic measure of entropy measured in bits, *p*_*j*_ refers to the fraction of the total effort devoted to event *e*_*il*_ by the *j*^*th*^ worker and *n*_*i*_ is the number of people on team *i*. This measure has the property that if *n*_*i*_ people contribute equal effort to recording an event, *c*(*e*_*il*_) = *n*_*i*_, whereas if a single person contributed all of the effort *c*(*e*_*il*_) = 1, hence it can be interpreted roughly as the effort-weighted number of collaborators per event. The team collaboration measure *c*_*i*_ for a particular team *i* is then *c*(*e*_*il*_) averaged over all events *e*_*il*_ worked on by that team.

**Fig 8:**
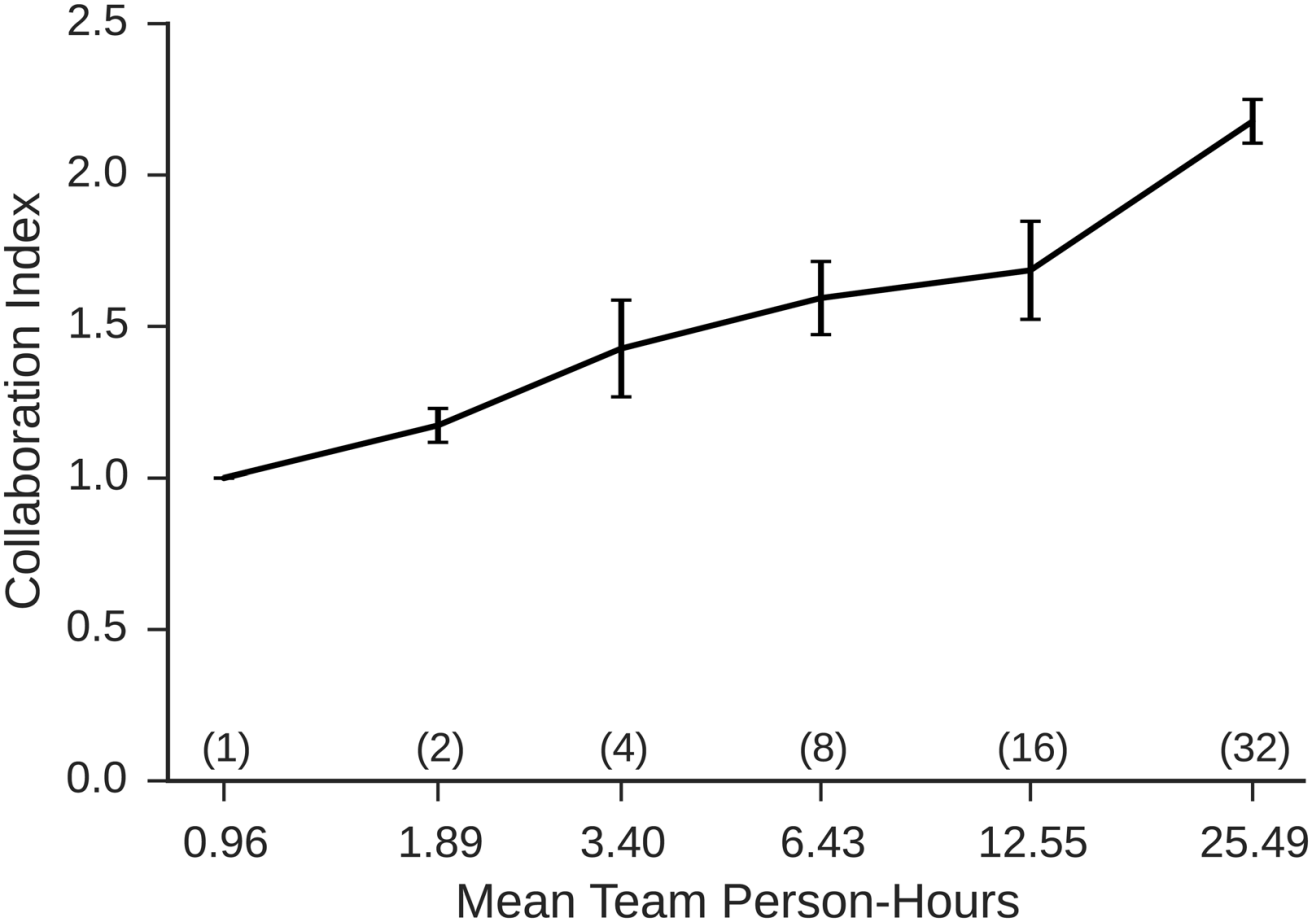
Collaboration activity, defined as the mean effort-weighted number of contributors per event, as function of person hours. Nominal group sizes are given in parentheses, error bars indicate one standard error, and the x-axis is on a log_2_ scale.

We note that although our measure of collaboration must by construction satisfy the inequality 1 ≤ *c*_*i*_ ≤ *n* it can take any intermediate value for any team size *n*. For example, individuals in teams of *n* > 1 could choose to work independently (i.e. on distinct event reports) in which case *c*_*i*_ = 1 regardless of *n*; thus values of *c*_*i*_ > 1 indicate collaboration on events beyond what is required by the task. Fig 8 shows collaborative effort *c*_*i*_ increased steadily as a function of team size, more than doubling for the largest teams relative to independent workers (ANOVA *F*(1, 45) = 98.77, *p* < 10^−10^). Fig 8 therefore complicates the picture presented in Figs 6 and 7: even as workers in larger teams exerted less effort individually, that is, and diverted their effort to less cognitively demanding sub-tasks, they also collaborated more, potentially allowing them to be more productive *collectively*.

### Evaluating the competing effects of effort and collaboration

The potentially countervailing effects of effort and collaboration mean that Fig 6 is unable to make a fair performance comparison between teams of different sizes: clearly a direct comparison unfairly benefits larger teams; however per-capita productivity, by failing to account for the potential benefits of collaboration, unfairly benefits smaller teams. Suppose, for example, that a handful of events are relatively easy to classify, perhaps because they are reported independently by many people, or because they involve very obvious and unambiguous damage, or because they refer to a well-known location. Conversely let us assume that the remaining events exhibit all the opposite attributes (few reports, obscure locations, ambiguous categories, etc.), and hence are relatively difficult to classify. In such circumstances, it is likely that all teams will first tackle the “easy” events and only when they are finished with those devote their energy to the more difficult ones. All else equal, therefore, per-capita productivity, measured solely in terms of number of correctly classified events, will be higher earlier on in the task when the easy events are being dealt with. Because they are working alone, independent workers encounter fewer events than the larger groups, and so are more likely to work on easy events, in which case their average productivity will be higher than for individuals large groups even if all workers are exerting equal effort. The flip side of this effect, however, is that the independent workers will also likely produce more redundant reports than workers in teams, who can see the work of their collaborators, and hence are in a better position to reduce redundancies and/or to correct errors. In this way, one might expect larger teams, even if less productive in terms of number of events classified per-capita, to produce fewer redundant reports (thereby increasing recall relative to an equivalent number of independent workers), or to produce more accurate reports (thereby increasing precision), or both.

**Fig 9:**
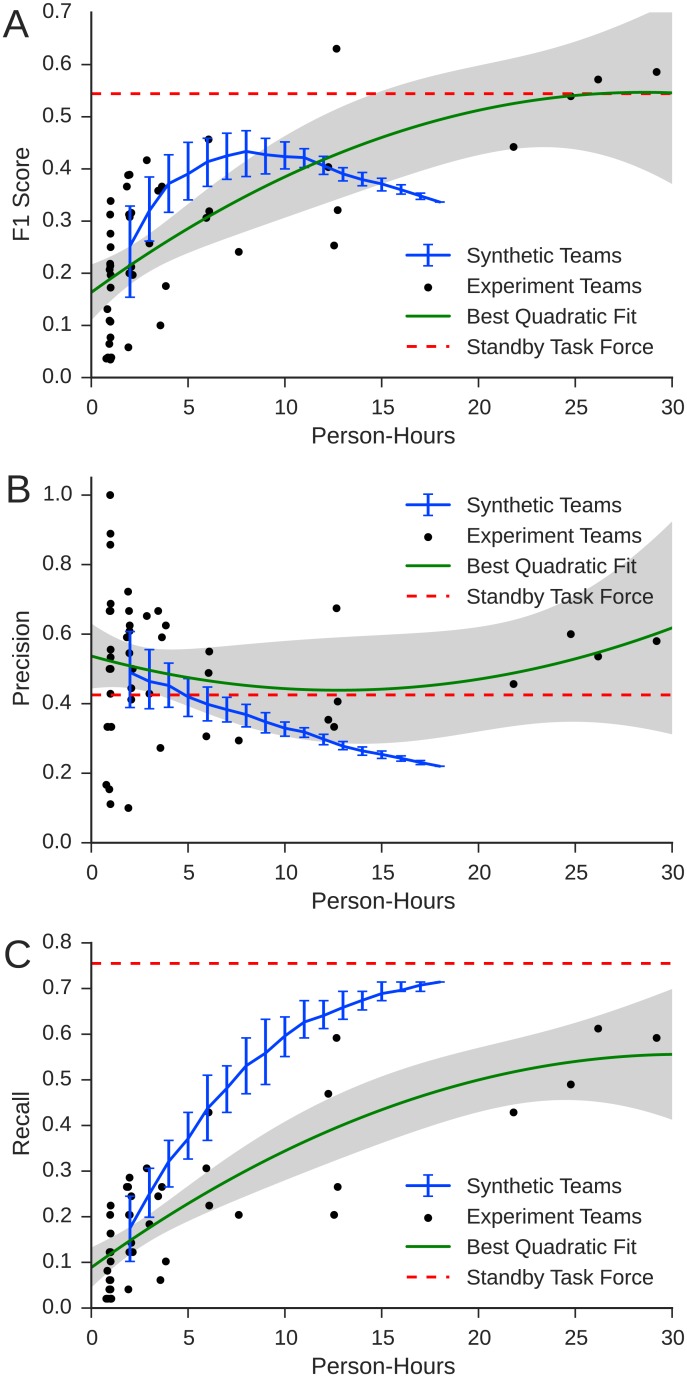
A: *F*_1_ score; B: recall; and C: precision of synthetic teams relative to actual teams. Solid blue line indicates mean performance of synthetic teams and error bars show the interquartile range. Dots correspond to the performance of experimental teams, solid green line corresponds to maximum likelihood fit of quadratic model, and shaded area corresponds to 95% confidence interval (as in Fig 6). Dashed red line shows the SBTF performance.

To account for the possibly countervailing effects of group size on productivity and coordination, we compare the output of the experimental teams with the collective output of *synthetic teams* of the same size, constructed by randomly sampling without replacement *n* ≤ 18 individuals from the total of 18 participants who had worked independently for one hour. Each such sample then induced a set of events generated by all individuals in that sample, for which we computed the associated precision, recall, and *F*_1_ score. We then repeated this process 100 times for all integer values of 2 ≤ *n* ≤ 16 as well as 18 times for *n* = 17 and once for *n* = 18 (the maximum number of distinct combinations for those values). Fig 9 shows the results, which suggest that for the largest teams the benefits of coordination were sufficiently large to outweigh any penalties associated either with the amount or allocation of individual effort. Fig 9A shows that for smaller values of person-hours synthetic teams generally outperformed real teams, consistent with the social loafing hypothesis. Interestingly, however, the performance of synthetic teams peaks around eight person-hours and then subsequently decreases. The result is that the larger experimental teams (*n* ≥ 16) significantly outperformed synthetic teams with *n* ≥ 16 on average (*t* = 5.77, *p* = 0.01; *μ*_exp_ = 0.535, *μ*_synth_ = 0.348). Moreover, the best-performing large team (one of the *n* = 16 teams) outperformed the best performing synthetic team size (*t* = −33.77, *p* < 10^−15^).

Fig 9B and 9C shed further light on this result: although recall for synthetic teams consistently dominates that of the experimental teams, precision decreases monotonically; hence the harmonic mean of precision and recall (i.e. *F*_1_ score) is non-monotonic. The opposing effects on precision and recall are of particular interest in light of our speculation (above) regarding how larger teams might benefit from collaboration vis-a-vis independent workers: on the one hand, because they are less likely than independent workers to report the same (i.e redundant) events; and on the other hand, because they are better able to correct errors in the events they report. Fig 9B and 9C suggest that both factors may be responsible. For any given event, that is, there is only one possible correct report whereas there are many possible incorrect reports. Combining the output of many independently generated reports, as we do for the synthetic teams, therefore increases the probability that at least one report will be correct (improving recall), but also increases the probability that at least one will be wrong (hurting precision). By allowing individuals to coordinate, as they do in teams, the differences between multiple conflicting reports can be reconciled and precision improved without much loss in recall.

## Discussion

Returning to our opening motivation, previous work has found *both* that individual effort diminishes with team size [19, 21] and also that individuals in teams benefit from collaboration [6, 34]. We emphasize, however, that neither existing theory nor prior experiments specify how these effects should combine to produce collective performance in a task of sufficient complexity to allow for both. The main immediate contribution of this work is therefore to show that for at least one complex and realistic task, namely crisis mapping, the positive effects of coordination dominate the negative effects of social loafing. Moreover, as we have emphasized earlier, crisis mapping shares many important features with other collective information processing activities, thus we expect our results to apply more generally to situations where collaboration can reduce redundancy and improve error checking.

Our results also open up a number of directions for future research. First, although social loafing has been studied extensively [19, 21], previous work has focused on relatively simple tasks (e.g. shouting, clapping) for which effort is one-dimensional. As we have shown here, for complex tasks where effort is multidimensional the same overall quantity of effort can be allocated across specialized activities, with potentially important consequences for performance. The social loafing result is also interesting because the compensation scheme explicitly discouraged free riding [9, 11]. As we noted earlier, individuals were clearly informed that their compensation would be proportional to their *own* activity, which we also informed them would be monitored; thus they had ample individual economic incentives not to free ride. As workers were also informed that their team’s performance would be compared only with other teams of the same size, simply knowing the size of the team to which they had been assigned should likewise not have encouraged free riding. That we nonetheless observed significant social loafing, both in terms of the total amount and also the allocation of effort, therefore suggests that relationship between economic incentives, task complexity, and group size in determining individual effort remains an open question that invites further experimental study.

Second, it is also interesting and somewhat puzzling that in our experiment we did not observe more of a positive effect of the division of labor [7]. One possible explanation is that although we trained all of our participants on the user interface prior to the experiment, we deliberately did not offer them guidance on how to organize themselves, and that the one hour allocated to the experiment did not allow sufficient time for a useful division of labor to emerge fully. Moreover, because we recruited inexperienced participants any specialization must necessarily have emerged during the experiment itself, hence again one hour may have been insufficient for individuals to learn particular roles and thereby fully realize the potential returns to specialization. For both these reasons we anticipate that future experiments, possibly running for longer or harnessing some combination of more experienced participants and more tailored instructions, will yield even larger benefits for teams.

Third, the surprisingly strong performance of our experimental teams relative to the actual Standby Task Force deployment for Typhoon Pablo allows us to make some tentative predictions about real world applications. To reiterate, the core SBTF deployment comprised between 18 and 32 volunteers with varying degrees of experience who produced a crisis map in roughly 12 hours using the same set of tweets as our participants. Although there were some differences between the 2012 deployment and our experiment that make an exact performance comparison problematic (see S1 Text), our experiments nonetheless demonstrated that a similar number of inexperienced crowd-workers using our platform could construct a map of comparable coverage in just one hour. These results suggest that with further optimization in the lab and possibly also field deployments of our platform in real crises, “always-on”, real-time crowdsourced crisis mapping may be possible, thereby significantly expanding the capacity of existing digital humanitarian organizations [35].

Finally, our mapping application and real-time experimental platform demonstrates that the high degree of interactivity, realism, and data instrumentation available in “virtual lab” settings is promising for understanding collective performance of large teams working on complex, real-world problems. We hope that future research will leverage this platform to study both crisis mapping and also other types of collaborative tasks, thereby addressing a broad range of questions about team performance including the role of experience [1], diversity [41], social sensitivity [42], network structure [6], and leadership [12].

## Supporting Information

S1 Text Comparison with SBTF Deployment. (PDF)

S2 Text Worker Inactivity. (PDF)

S3 Text Performance Metrics. (PDF)

S4 Text Quadratic Model Fits. (PDF)

S5 Text Consent Form. (PDF)

S6 Text Crisis Mapping Tutorial. (PDF)
