# Supplementary material for: An Experimental Study of Team Size and Performance on a Complex Task
Source: PLoS One. 2016 Apr 15;11(4):e0153048. doi: 10.1371/journal.pone.0153048 (PMC4833429; doi:10.1371/journal.pone.0153048)
Supplement: S1 Text — (PDF) [file pone.0153048.s001.pdf]

Comparison with SBTF Deployment

As emphasized in the main text, an important differentiating feature of our experimental design was its close resemblance with the real-world version of crisis mapping, in particular the Standby Task Force's Dec 2012 deployment for Typhoon Pablo. Nevertheless, our study differed from the SBTF deployment in certain respects, which we detail here.

- The SBTF worked during the deployment using a spreadsheet instead of CrowdMapper. As noted in the main text, the spreadsheet was organized around tweets containing relevant information and did not attempt to de-duplicate similar damage reports in the same area, resulting in many event reports in heavily damaged areas. This difference results in a lower precision in our scoring algorithm relative to the teams in our experiment, of which some attempted to de-duplicate event reports.
- Because of the length of time between the original deployment (Dec 2012) and our experiments (Aug 2014), a small number of the links that were used by the SBTF were no longer usable by our teams. As a consequence it is conceivable that the SBTF could have correctly reported events that could not have appeared in our gold standard, and that these events would be registered by our scoring methods as errors. Although manual inspection of the SBTF data reveals that these events were extremely rare in practice, their presence could again have reduced the apparent precision of the SBTF.
- The SBTF workforce comprised volunteers in a crisis situation who might have differed considerably in disposition and motivation from paid participants in a simulated exercise.
- In contrast with our participants, SBTF volunteers all received some degree of mapping training, and were organized by experienced leaders who had worked on previous deployments.
- Our experimental platform integrated all the mapping and communication functions into a single application, greatly reducing the overhead compared to working in several applications (e.g. Skype, Google Docs, Google Spreadsheets, and other websites) simultaneously.
- Our experiments required participants to be online at the same time and thus improved the possibilities of coordination between multiple people.

For all these reasons the performance comparison between the SBTF and our experimental groups should be regarded as approximate, not exact. We also note, however, that the differences do not convey a consistent benefit on either population: on the one hand, the leadership and training afforded SBTF workers would appear to advantage them with respect to our subjects; but on the other hand, the integrated Crowdmapper platform and simultaneous recruiting used in our experiment may have benefit our subjects with respect to the SBTF volunteers. In the absence of any large systematic advantage, therefore, we conclude that the surprisingly comparable performance of our the real and experimental populations is a persuasive indicator of external validity for our design.
