# Supplementary material for: An Experimental Study of Team Size and Performance on a Complex Task
Source: PLoS One. 2016 Apr 15;11(4):e0153048. doi: 10.1371/journal.pone.0153048 (PMC4833429; doi:10.1371/journal.pone.0153048)
Supplement: S2 Text — (PDF) [file pone.0153048.s002.pdf]

Worker Inactivity

We monitored worker activity using software in the browser that keeps track of the time elapsed since the user's last mouse click or keystroke (an "activity"). If the user does not perform any activity for a specified threshold time $\hat{\tau}$, then the span of time since the last activity will be retroactively marked as idle. Users who are marked as idle are not paid for this time, and both the idle state and current range of potential earnings were displayed to workers in real-time. We used a threshold of $\hat{\tau} = 8$ minutes for this experiment, since our expectation was that users would be clicking on links and visiting other websites during the experiment, thus may appear inactive even while working in a different browser window. Our pilot studies indicated that 8 minutes was ample time for them to return to the main app without being penalized, and we recorded only a handful of idle periods during the experiments themselves. Finally, we did not receive any complaints from users to the effect that they had been timed out while trying to work; thus we believe our system was effective in enforcing the incentive system that we designed without unfairly penalizing active workers.
