# Supplementary material for: An Experimental Study of Team Size and Performance on a Complex Task
Source: PLoS One. 2016 Apr 15;11(4):e0153048. doi: 10.1371/journal.pone.0153048 (PMC4833429; doi:10.1371/journal.pone.0153048)
Supplement: S3 Text — (PDF) [file pone.0153048.s003.pdf]

Performance Metrics

As described in the main text, we computed three performance measures: *precision*, defined as the fraction of events identified by team i that matched the gold standard; *recall*, defined as the fraction of all events in the gold standard that team i correctly identified; and F_1 score, defined as the harmonic of mean of precision and recall. In more detail, the metrics were computed as follows. First for each event e_{il} found by team i and each event e_g in the gold standard, we define the *fractional score*

$$s(e_{il}, e_g) = \frac{1}{4}I(\text{correct type}) + \frac{1}{4}I(\text{correct province}) + \frac{1}{4}I(\text{correct region}) \\ + \frac{1}{4}\log_{10}(\max(10\text{ km}, \min(100\text{ km}, \text{distance in km})) - 1) \tag{1}$$

$s(\cdot)$ takes on a value between 0 and 1 by equally scoring the the type, province, region, and location fields; location receives full score for a distance within 10 km, and none beyond 100 km.

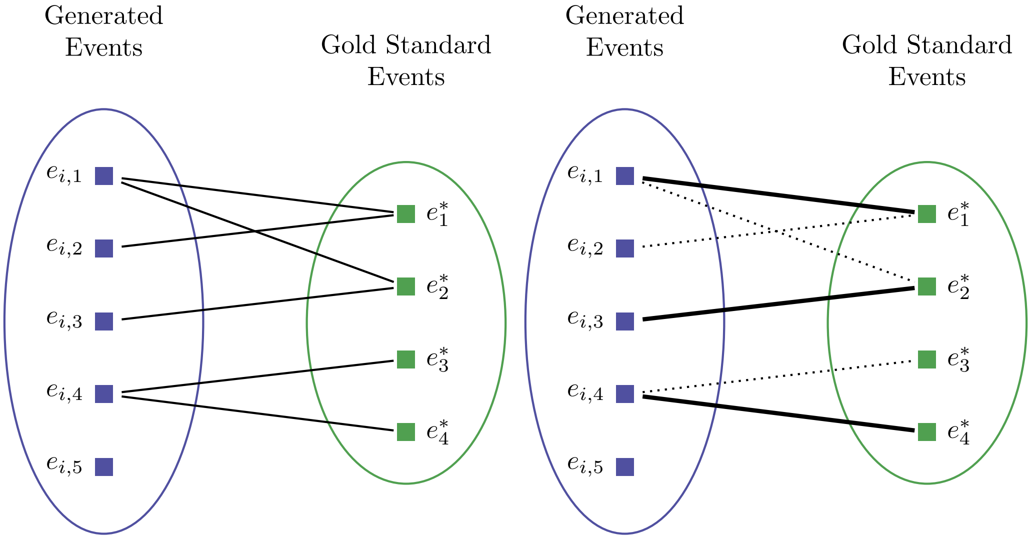

Figure 1: Illustration of the matching algorithm. **A**: Scores $s(e_{il}, e_g) \geq 2/3$ induce edges in an unweighted bipartite graph. **B**: A maximum matching of this graph, with weight 3. In this case, the three events $e_{i,1-3}$ can only count for two events in the gold standard, so one is redundant. Although the event $e_{i,4}$ is close enough to match two events in the gold standard, it can only count for one. The precision is 3/5 and the recall is 3/4.

Next, as illustrated in Fig. 1A, we constructed an unweighted bipartite graph in which the events e_{il} reported by team i constituted one node set (blue nodes on the left), the gold standard events e_g constituted the other set (green nodes), and edges exist between e_{il} and e_g when $s(e_{il}, e_g) \geq 2/3$. We chose this threshold such that in order to be scored "correct" an event would need either to have all three categorical fields correct, or else two categorical fields correct and be within approximately 20 km of the gold standard latitude-longitude. We then computed the number of correct events as the size of the *maximum matching*[1] of the resulting graph. As illustrated in Fig. 1B, the maximum matching effectively gives teams credit for any event that is "close enough" to the original (i.e. exceeds the 2/3 threshold), while preventing duplicate

events from counting twice. Finally, we computed precision p_i, recall r_i, and F_1 score f_i for each team i as

$$p_i = \frac{|\{\text{gold standard event records}\} \cap \{\text{identified event records}\}|}{|\{\text{identified event records}\}|},$$

$$r_i = \frac{|\{\text{gold standard event records}\} \cap \{\text{identified event records}\}|}{|\{\text{gold standard event records}\}|}, \text{ and}$$

$$f_i = 2\frac{r_i \cdot p_i}{r_i + p_i}$$

respectively, where the numerator for p_i and r_i is the size of the maximum matching.

References

[1] Harold W Kuhn. The hungarian method for the assignment problem. *Naval research logistics quarterly*, 2(1-2):83–97, 1955.
