# Supplementary material for: An Experimental Study of Team Size and Performance on a Complex Task
Source: PLoS One. 2016 Apr 15;11(4):e0153048. doi: 10.1371/journal.pone.0153048 (PMC4833429; doi:10.1371/journal.pone.0153048)
Supplement: S4 Text — (PDF) [file pone.0153048.s004.pdf]

Quadratic Model Fits

As mentioned in the main text, we fit the following quadratic model for team performance as a function of team size (approximated by person-hours ω_p):

$$y \sim \beta_0 + \beta_1 \omega_p + \beta_2 \omega_p^2 + \epsilon$$

The parameters of the model for the dependent variables of F_1 score, precision, and recall are given in Tables 1, 2 and 3, respectively.

Table 1: F1 Score

	Dependent variable:
	F_1 score
β_0	0.263***
	(0.016)
β_1	0.726***
	(0.113)
β_2	−0.163
	(0.113)
Observations	47
R^2	0.498
Adjusted R^2	0.476
Residual Std. Error	0.113 (df = 44)
F Statistic	21.857*** (df = 2; 44)
Note:	*p<0.1; **p<0.05; ***p<0.01

Table 2: Precision

	Dependent variable:
	Precision
β_0	0.505***
	(0.029)
β_1	−0.005
	(0.197)
β_2	0.209
	(0.197)
Observations	47
R^2	0.025
Adjusted R^2	−0.019
Residual Std. Error	0.197 (df = 44)
F Statistic	0.563 (df = 2; 44)
Note:	*p<0.1; **p<0.05; ***p<0.01

Table 3: Recall

	Dependent variable:
	Recall
β_0	0.205***
	(0.013)
β_1	0.870***
	(0.092)
β_2	−0.173*
	(0.092)
Observations	47
R^2	0.678
Adjusted R^2	0.663
Residual Std. Error	0.092 (df = 44)
F Statistic	46.231*** (df = 2; 44)
Note:	*p<0.1; **p<0.05; ***p<0.01
