# Supplementary material for: An Experimental Study of Team Size and Performance on a Complex Task
Source: PLoS One. 2016 Apr 15;11(4):e0153048. doi: 10.1371/journal.pone.0153048 (PMC4833429; doi:10.1371/journal.pone.0153048)
Supplement: S5 Text — (PDF) [file pone.0153048.s005.pdf]

Consent Form

Below is the full text of the form used to solicit informed consent from all participants in the crisis mapping experiment.

MICROSOFT RESEARCH CRISIS MAPPING PROJECT
PARTICIPATION CONSENT FORM

INTRODUCTION:

Thank you for deciding to volunteer in a Microsoft Corporation research project. The research project will consist of one session to assess how teams of people can work together on a crisis-mapping problem. Please note that you have no obligation to participate and you may decide to terminate your participation at any time. Also note that Microsoft has no obligation to disclose any research results. You understand that the researcher has the right to withdraw you from participation in the project at any time. Below is a description of the research project, and your consent to participate. You are asked to read this information carefully. If you agree to participate, check the box provided on the project website to indicate that you have read and understood the information furnished on this consent form.

PROCEDURES:

During this project, you will be shown Twitter reports from a historical natural disaster (e.g. hurricane, typhoon, earthquake, etc.) This natural disaster and the data you see is from the past, and is not an actual current crisis. Together with your assigned team, you must figure out which reports describe relevant crisis events. You must then record these events including by describing the type of event, its description, and its location. The task will last for the amount of time specified in the tutorial, usually about one hour. At the end of the project we will ask you some questions about yourself and your experience during the project. We will also ask you for your permission to be contacted by us about future projects that we may conduct, whether related to crisis mapping or to other projects that we think may be of interest to you. You are under no obligation to answer these questions or to provide your permission, and your compensation will not be affected by your answers.

COMPENSATION:

Your compensation, in the form of a bonus paid on Amazon Mechanical Turk, is based on your teams relative performance. Bonuses will be calculated as: (Minutes You Worked)/60 [Base Rate + Team Bonus]. Your Team Bonus will be calculated as: [Bonus Rate (Number of valid events your team correctly records) / (Highest number of valid events labeled by any team)]. The Base Rate and Bonus Rate are noted in the tutorial. For example, if the Base Rate is $6 and the Bonus Rate is $9, you will earn from $6 to $15 for one hour of work, depending your teams performance. Note that if you quit early or pause the task, you will only be paid for the fraction of time that you worked.

PERSONAL INFORMATION:

During the project we may need to collect personal information about you such as your city/town, age, marital status, occupation, education, ethnicity, etc. The personal information collected during this project will be used to perform research. Except as otherwise described in this statement, personal information you provide during this project will not be shared outside of Microsoft and its controlled subsidiaries and affiliates without your permission.

RESEARCH DATA & FEEDBACK:

You give your permission to Microsoft to record or otherwise document and collect information about your participation in the research project in any format and medium (Data). Microsoft shall own all Data in connection with the research project. You may also provide suggestions, comments or other feedback (Feedback) to Microsoft with respect to the research project. Feedback is entirely voluntarily, and Microsoft shall be free to use, disclose, reproduce, license, or otherwise distribute, and exploit the Feedback and Data.

CONFIDENTIALITY:

The research project is confidential and you agree to: (a) never disclose it to anyone else (accept in accordance with a judicial or other governmental order); and (b) keep it secret as you would your own confidential information. Microsoft does not consider all information about the research project to be confidential. If you legally received information from someone other than Microsoft, and that person was entitled to share the information with you and did not obligate you to keep it a secret, you do not need to

keep that information secret. The same holds true for information Microsoft disclosed to you or someone else without obligation to keep it secret or information that you develop independently.

YOUR AUTHORITY TO PARTICIPATE:

You represent that you have the full right and authority to agree to this consent form, and you will not disclose to Microsoft any non-public information, whether yours or a third partys without notifying Microsoft in advance. **YOU ASSUME THE FULL RISK OF ANY INJURIES, DAMAGES, OR LOSSES YOU MAY SUSTAIN AS A RESULT OF YOUR PARTICIPATION IN THE PROJECT. IN ADDITION, YOU AGREE TO RELEASE AND HOLD HARMLESS MICROSOFT AND ITS AFFILIATES FROM ANY AND ALL CLAIMS THAT YOU MAY HAVE NOW OR IN THE FUTURE RELATED TO OR ARISING FROM YOUR PARTICIPATION IN THE RESEARCH PROJECT, AND YOU HEREBY WAIVE ALL SUCH CLAIMS. MICROSOFT WILL NOT BE LIABLE FOR ANY DAMAGES RELATED TO YOUR PARTICIPATION IN THE PROJECT.**
