# Supplementary material for: An Experimental Study of Team Size and Performance on a Complex Task
Source: PLoS One. 2016 Apr 15;11(4):e0153048. doi: 10.1371/journal.pone.0153048 (PMC4833429; doi:10.1371/journal.pone.0153048)
Supplement: S6 Text — (PDF) [file pone.0153048.s006.pdf]

Crisis Mapping Tutorial

The tutorial was presented as an interactive step-by-step interface requiring participants to complete each step below, many of which required interaction with the mapping tool itself. Figure 1 shows a screenshot of the tutorial as an example. The text of the tutorial below is adapted from the web-based HTML version, which included icons and additional formatting and is available at `https://github.com/mizzao/CrowdMapper`.

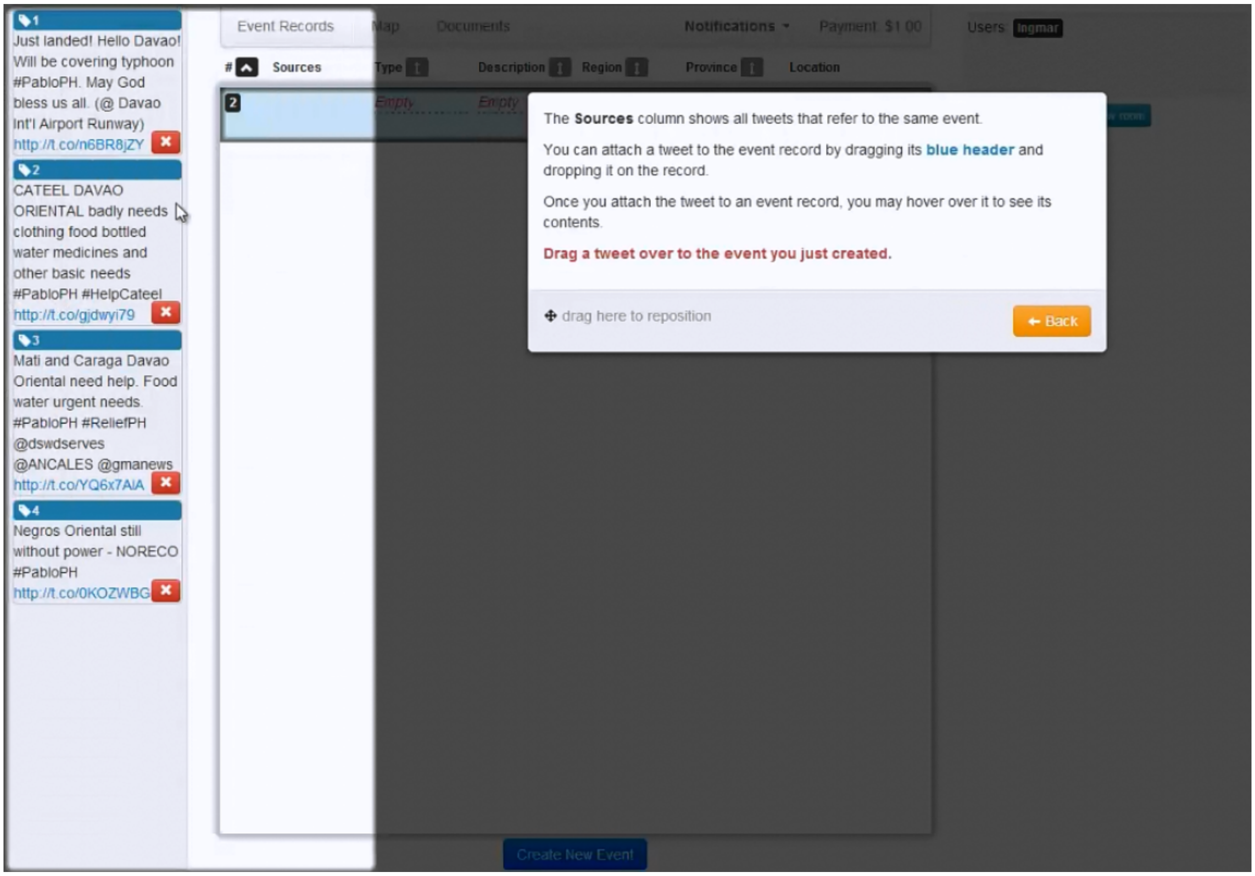

Figure 1: One step of the interactive tutorial. In this step, the user was required to drag and drop a tweet from the list on the left into an event record.

Welcome to the Crisis Mapping Project, a Microsoft Corporation research project.
Before beginning, you will complete a brief tutorial to familiarize yourself with the Crisis Mapping interface.
You would have completed this tutorial when you registered for the Crisis Mapping Project, but this will refresh your memory.
To learn more about the team conducting this research, please visit the following links:

- `http://research.microsoft.com/en-us/teams/oess_nyc/`
- `http://www.andrewmao.net/`

For the best experience, please maximize the window containing this task or make it as large as possible.

We need your help to better understand how teams of people can work together on a crisis mapping problem.
A "crisis map" is created in response to a natural or humanitarian disaster by monitoring social media for reports of events on the ground and placing them on a map.

In this project, you will work with a team of other crisis mappers to create a crisis map for Typhoon Pablo, a Category 5 hurricane which hit the Philippines in December 2012.
NOTE: This hurricane and the data you see is historical. This is not an actual current crisis.

To create the crisis map, you and your team members will be shown actual Twitter reports from Typhoon Pablo. Your team must figure out which reports describe relevant crisis events.
In collaboration with your team, you must record these events describing the types of events, their descriptions, and their locations.
Relevant reports include damage to infrastructure, reports of flooded regions, casualties, local residents that have been displaced, and evacuation centers. These should be recorded on the crisis map.
Other reports are irrelevant or inaccurate and should not be recorded.

This tutorial will provide a general description of the task and explain the basic functions of the Crisis Mapping tool, but it is your job to figure out how to solve the problem.
By effectively solving this problem and creating an accurate crisis map with your teammates, you can help us learn how to better respond to future natural disasters.
In some cases, you may have a small team, or be the only *person on your team, and you may not be able to finish recording events for all of the reports. You should focus on creating accurate, verifiable records over incomplete or partially categorized events.*

Data reports appear as Tweets in the Stream on the left.
This tutorial shows a few pieces of example data. You will see much more actual data in the task.

You should click on links to see what reports refer to, and to determine whether or not the data is relevant.
You may search the Internet for places and phrases mentioned in reports.
You may also hide tweets that appear to be irrelevant by clicking the red **X** button. When you hide a tweet, it disappears for everyone.
To continue, find one irrelevant tweet and hide it.

The navbar allows you to access the three primary ways to record information and work with others: *event records*, *map*, and *documents*.
It also shows *notifications* you receive from other mappers.

The *Event Records* pane is the primary tool for recording events.
Here you will see all of the event records you and your collaborators create.

Event records comprise several fields describing information about events.
Your job is to fill in these fields for each relevant crisis event.
Some information about the event can be found in the tweet itself, while other information can be found online or will require your own judgment.

Create a new event by clicking the "Create New Event" button at the *bottom of the page.*
Create a new event now.

After you create an event, you will automatically be placed in edit mode.
You may also edit an existing event record by double-clicking or by clicking the blue (pencil) button. You will see edits by other mappers in real-time.
Only one person can edit an event record at a time, so when you're done, click the green (Save) button to save the event.

The first column shows records numbered in the order they were created.
You can use this number to coordinate with other mappers in the chat room, which we'll show later.

The *Sources* column shows all tweets that refer to the same event.
You can attach a tweet to the event record by dragging its *blue header* and dropping it on the record.
Once you attach the tweet to an event record, you may hover over it to see its contents.
Drag a tweet over to the event you just created.

Events that are relevant for crisis mapping will fit into one of these types:

- Damaged bridges
- Damaged crops
- Damaged hospitals/health facilities
- Damaged housing
- Damaged roads
- Damaged schools
- Damaged vehicles
- Damaged infrastructure (other)
- Death(s) reported
- Displaced population
- Evacuation center
- Flooding

When you are editing an event record, you can *click a field* to enter a value. Fields that are empty will display the text (empty).
Classify the event by clicking the *Type* field and choosing from the dropdown menu.

The *Description* column allows you to record a brief description of the event.
Write something in the event's description.

The *Region* column shows the region where the event occurred.
The Philippines has 17 administrative regions. You may need to look for regions and cities on other websites if you can't find them directly on the map.
Add your best guess of the region from the tweet you just attached. Hover your mouse over the tweet you just attached to see its text.

The *Province* column is the province where the event occurred.
There are 80 provinces in the Philippines.
If you can find the province for the tweet, enter it in now.

The *Location* column shows the longitude/latitude for the event.
If an event record does not have a location yet, you can place it on the map. We will show you how to do this momentarily.
If you find these numbers from a map service on the Internet, you can use these numbers while editing the event.

You may also *add additional tweets to existing event records* if they refer to the same event. You can also *drag tweets from one event to another* to correct mistakes or reorganize records.
If you *hover over an attached tweet* and find it to be irrelevant, you can use the (red X) to hide it.
If you can find another tweet that refers to the same event as this one, add it to the record by dragging and dropping.

You can sort events by their data by clicking the arrows in the event header.

The *Map* displays a marker for each recorded event.
If no longitude/latitude has been entered for an event, the marker will not appear on the map.

Use these map controls to pan around the map, and zoom in and out. You can also scroll and drag with your mouse.<br>With your mouse, you can also pan by dragging and zoom by scrolling.<br>*This map is limited to the area around the Philippines, so you will need to first zoom in before you can pan the view.*
You can press the (Locate) button to place an event on the map. You may need to search on the Internet to find a precise location for an event that you see.<br>As you move your mouse over the map, note that the coordinates are shown in the *bottom right.* Use this to place the event in a precise location.<br>**Using the location information from the tweet, place the event on the map.**
When looking at the map, you may hover or click on any event to view its details, and drag any event marker to change its geographical location.<br>As you learn more information about an event, you will be able to pinpoint its location more accurately.
Notice that the longitude/latitude has automatically been updated in the event record. Now, you can also edit the coordinates by clicking them directly.<br>Now, select the province, if you know it, and save the record using the (Save) button. This will let someone else edit the event.<br>**Save the event now.**<br>Once you have saved the event, you may click its location to see it on the map.
You may also check the accuracy of the events records created by other crisis mappers by verifying their data. The (checkbox) area shows that no one has verified this event yet.<br>If you think an event record is accurate, you may verify it by hovering over this area and clicking the (Verify) button. You can also remove your vote for an event if you think it is no longer accurate.<br>**Since you created this event and believe it is accurate, check it now.**
Documents are available for recording information relevant to the task but not included in event records. They are shared with everyone else and can be edited simultaneously.<br>**Create a document by clicking the *New Document* button.**
You can rename any document by clicking on its title and create new documents as necessary.<br>**Type something in the document.** Other mappers will see this when they read the document.<br>When you're done typing, continue to the next step.
The *User List* shows team members who are currently online in (green), and those who have stopped participating in (grey). You are shown in (black).<br>When other users are online, you may hover your mouse over their name to invite them to chat.<br>*If you are working by yourself, you will see only your own name here.*
You may create and join *chat rooms* to communicate with other users. Use the (New room) button to create a chat room.<br>**Create a new chat room now.**
When other users invite you to chat or mention you in a chat room, you will see notifications appear here. Click on them to go to the chat room.
You will see a list of all chat rooms and the number of people in them, but you can only be in one chat room at a time.<br>To join a chat room, just click on it.
When you are in a chat room, you can see who else is in that room. Each chat room displays the entire history of chats in that room.<br>To leave a chat room, you can either click on "leave" or click on another chat room, which will then become active.

When you are in a chat room, you can use special characters to notify users and reference tweets or events.

- Use @ to mention a user by name. This will notify them.
- Use (tilde) to reference a tweet by its number.
- Use # to reference an event by its number.

When you start typing the symbols above, an automatic filtered list will appear.
Selecting an item in this list will create a link in your chat message that users can click on to jump to the tweet or event.
Type something in the chat. For example, to tell another user to look at the event you created, type "@myself can you take a look at event #1?" You are talking to yourself here, but you will be chatting with other users in the actual task.

The project will run for one hour.
You may quit the project at any time.
If you perform no activity for several minutes, your session will be automatically idled.

Your payment will be determined by how much time you personally spend working on the task, and also the overall performance of your team.
Your team may be competing with other teams, and your team's performance will be measured relative to either the best-performing team or other benchmarks. Your compensation will be calculated as:
(minutes you worked)/60 × ($6 + your team bonus)
Your team bonus will be calculated as:
(Valid events your team correctly records)/(Highest number of valid events labeled by any team)
If you work for the full one hour, your compensation will be no less than $6 and as much as $15, depending on your team's performance.
Your payment will be shown during the task in the highlighted region. If you quit early or are idle you will only by paid for the fraction of time you work.
Thus, you should not just work hard yourself, but also help your team succeed.
Sometimes, you may be on a team by yourself. In this case, you will be evaluated against others who are also working by themselves.

Please read and acknowledge the *terms of use* for participating in this project.
Click to view the terms of use. (text of Section **??**)
By clicking the checkbox below you confirm that you are at least 18 years of age, and that you understand what the project is about and how and why it is being done.
Some tweets may contain descriptions or images of human or animal suffering, and may be disturbing to some users. If you know that you are adversely affected by this kind of content, feel free to exit the experiment.
By checking the box, you also acknowledge that you accept this possibility through your participation in crisis mapping.
(checkbox) I have read and acknowledged the terms of use.
If you have any questions about this project, please contact Andrew Mao at *[email].*
Click 'Finish' below to join a team of other workers and start crisis mapping!
